# Cyanide overproduction impairs cellular bioenergetics in Down syndrome

**DOI:** 10.1016/j.neurot.2025.e00719

**Published:** 2025-08-18

**Authors:** Maria Petrosino, Karim Zuhra, Anna Kieronska-Rudek, Lucia Janickova, Olivier Bremer, Moustafa Khalaf, Brian A. Logue, Csaba Szabo

**Affiliations:** a Section of Pharmacology, Department of Oncology, Microbiology and Immunology, Faculty of Science and Medicine, University of Fribourg, Fribourg, Switzerland; b Department of Chemistry, Biochemistry and Physics, South Dakota State University, Brookings, SD, USA

**Keywords:** Mitochondria, Bioenergetics, Gasotransmitters

## Abstract

Cyanide exerts its toxic effects primarily by inhibiting mitochondrial Complex IV (Cytochrome *c* oxidase, CCOx). Recent studies have shown that mammalian cells can endogenously produce cyanide from glycine via a lysosomal pathway. At low concentrations, cyanide may play regulatory roles, but at higher levels, it causes metabolic inhibition. Here we show that Down syndrome (DS) cells and tissues exhibit significant overproduction of cyanide, contributing to cellular metabolic suppression. DS rats show elevated blood cyanide levels, and their tissues generate more cyanide than wild-type controls—both under basal conditions and following glycine supplementation. Similarly, human DS fibroblasts produce higher levels of cyanide than healthy control cells. We attribute this increased cyanide production in DS to the marked downregulation of thiosulfate sulfurtransferase (TST, also known as rhodanese), the key enzyme responsible for cyanide detoxification. Importantly, suppression of lysosomal cyanide production in DS cells (through cyanide scavengers, lysosomal deacidification, or inhibition of serine/glycine conversion) improves cellular bioenergetics and/or enhances cell proliferation rates. Previous work has implicated excessive hydrogen sulfide (H_2_S) production, another endogenous gaseous signaling molecule that inhibits CCOx, in DS-associated metabolic suppression. Our current findings indicate that cyanide overproduction may also contribute to this dysfunction. Cyanide and H_2_S may act cooperatively on the same molecular target. These results open the possibility of developing therapeutic strategies that target cyanide or both cyanide and H_2_S to counteract DS-associated metabolic impairment.

## Introduction

Down syndrome (DS) is a common genetic condition characterized by an additional—either full or partial—copy of chromosome 21. Individuals with DS exhibit distinct morphological features and various biochemical alterations and maladaptations [1].

Mitochondrial respiration is essential for maintaining cellular ATP levels in eukaryotic cells. In DS, however, there is a notable metabolic shift characterized by significant suppression of mitochondrial ATP generation and a compensatory increase in glycolysis, a less efficient bioenergetic process. Evidence for this metabolic shift was found using glucose fluxomic studies in human DS cells [2] and further confirmed by a meta-analysis of metabolomic datasets [3]. This impairment of mitochondrial ATP production in DS (a phenomenon sometimes termed as “pseudohypoxia”) is proposed to contribute significantly to neurodevelopmental and neurocognitive deficits observed in DS [3].

Cyanide is a well-known cytotoxic agent, recognized for inhibiting mitochondrial electron transport through binding to the heme a3 group in mitochondrial Complex IV (cytochrome *c* oxidase, CCOx) [4]. Although evidence over the past two decades has suggested that endogenous cyanide production may occur in mammalian cells and tissues [5,6]. However, the mammalian cyanide-generating pathway has only recently been characterized in detail [7]. Cyanide is produced from glycine within lysosomes under acidic conditions, and its cellular levels are tightly regulated by the mitochondrial enzyme thiosulfate sulfurtransferase (TST or rhodanese) [7]. At low concentrations, cyanide exerts physiological regulatory and protective effects; however, its overproduction causes metabolic suppression through inhibition of CCOx [[7], [8], [9]].

The above results open the door for investigating the various potential regulatory roles of cyanide in health and disease. Given **(a)** the significant evidence of the development of pseudohypoxia (i.e. inhibition of mitochondrial electron transport and suppression of aerobic ATP generation) in DS [2,3] and **(b)** the well-known ability of cyanide to inhibit mitochondrial function [4], we explored the possibility that endogenous cyanide generation or overproduction may contribute to the DS-associated metabolic dysfunction. Prior studies have suggested that overpoduction of the endogenous mammalian gaseous transmitter hydrogen sulfide (H_2_S) may be, at least partly, responsible for these actions [2,10]. Here we demonstrate that significant cyanide overproduction occurs in DS cells and tissues, and present data that indicate that endogenous cyanide biogenesis contributes to the DS-associated cellular bioenergetic and functional impairment. Furthermore, our results suggest that endogenously generated cyanide and H_2_S may contribute to these alterations in a cumulative or cooperative manner.

## Methods

### Animals

The rat tissues used in the experiments were collected from the transgenic Sprague-Dawley Dup (Rno20)Yah rat model of DS, containing the duplication of Rno20 [11] as well as from age-matched wild-type control rats. This model features a duplicated segment of rat chromosome 20 (Rno20) comprising 74 genes in the Umodl1–Prmt2 region, which includes the *cbs* gene. Thus, this model lacks duplication of some rat orthologs of human chromosome 21 genes; importantly, unlike many mouse DS models, it also does not duplicate any genes beyond those present on human chromosome 21. The functional characterization of this rat model has so far been reported in only one study [11]. In that study, Dup (Rno20)Yah DS rats displayed no significant developmental defects in the brain or heart and showed normal locomotion and exploratory behavior. They exhibited no impairment in spatial working memory formation (T-maze paradigm) but did show impaired recognition memory (novel object recognition test) and a reduced preference for social novelty [11]. They also exhibit significant alterations in brain EEG activity, and –– in line with the gene dose effect of cbs, exhibit higher CBS expression in the brain than wild-type controls [11]. The animals were 3–8 months old, and the control animals were littermates of the DS rats. The breeding colony was maintained by pairing heterozygous rats with wild-type rats of the same genetic background. Rats were housed in a temperature-controlled room (21 ​± ​2 ​°C) in individually ventilated cages (IVC) with fine bedding, standard nesting material, and a standard cardboard tunnel for enrichment. Animals were maintained in a 12 ​h light/dark cycle and had ad libitum access to food and water. All animal handling was performed in accordance with the “Swiss Federal Animal Welfare Act of December 16, 2005”.

### Sample preparation: rat blood collection

Animals were euthanized via i.p. injection of pentobarbital at a dose of 300 ​mg/kg. Once the animal no longer responded to noxious stimuli, a midline abdominal incision was performed, followed by removal of the thoracic cage to expose the heart. Using a 1 ​ml syringe fitted with a 26G needle, the right ventricle was punctured, and blood was drawn gently to avoid ventricular collapse. Care was taken to orient the bevel of the needle outward (i.e., away from the interventricular septum) to prevent damage and ensure proper blood flow. After removal of the needle, the collected blood was transferred into an EDTA-containing microtube (1.3 ​ml ​K_3_E Micro Tube, Sarstedt, Ref: 41.1504.005), gently mixed to ensure proper action of the EDTA and kept on ice. From the collected 1 ​ml blood samples, 500 ​μl was transferred into 500 ​μl cryotubes for flash freezing and stored at −80 ​°C until further analysis of cyanide levels using the Cyanalyzer LC–MS/MS method [7] and the other 500 ​μl was used for preparation of plasma and detection of additional metabolites (see below).

### Sample preparation: rat tissue collection

Animals were euthanized by intraperitoneal injection of pentobarbital at a dose of 300 ​mg/kg, followed by exsanguination. To remove residual blood from the tissues, the animals were perfused via the ascending aorta with 60 ​ml of chilled phosphate-buffered saline (PBS) for 2–4 ​min. Liver, brain and skeletal muscles were collected, snap-frozen in liquid nitrogen and stored at −80 ​°C until further analysis.

### Cell culture

Fibroblasts obtained from healthy control individuals were obtained from: CCD-1064Sk (ATCC® CRL-2076™), GM05756 (Coriell Institute), #286 (Lejenue Institute, Paris, France), GM5659 (Coriell Institute, Camden, NJ, USA), Detroit 551 (ATCC® CCL-110™), GM08447 (Coriell Institute), GM0041 (Coriell Institute), #368 (Lejenue Institute). Fibroblasts obtained from DS individuals were obtained from: AG07096 (Coriell Institute), #285 (Lejenue Institute), AG05397 (Coriell Institute), #532 (Lejenue Institute), Detroit 539 (ATCC® CCL-84™), AG04616 (Coriell Institute), GM2571 (Coriell Institute), #369 (Lejenue Institute). HepG2 hepatocellular carcinoma cells (American Type Culture Collection (ATCC) HB-8065) were used for mitochondrial Complex IV analysis to test the effect of CN and H_2_S donation. HepG2 cells were grown in DMEM containing 1 ​g/l glucose (Gibco, #21885) supplemented with 10 ​% FBS and 100 units per ml penicillin and 100 ​μg/ml streptomycin. Fibroblasts were grown in Adv. DMEM/F12 (Gibco, #12634) medium, supplemented with 10 ​% FBS, GlutaMAX (2 ​mM), lactalbumin hydrolysate (0.1 ​%) and 100 units per ml penicillin and 100 ​μg/ml streptomycin. All cells were incubated in humidified incubator at 37 ​°C and 5 ​% CO_2_ atmosphere and for experiments and sub-culturing, cells were rinsed with PBS and detached from T75 flasks by incubating with 0.25 ​% (w/v) trypsin containing 0.53 ​mM EDTA for 2–5 ​min at 37 ​°C followed by resuspension in culture medium. For the measurement of cyanide generation, CTR or DS fibroblasts were seeded in 6 well plates (300,000 ​cells/well) and incubated at 37 ​°C in humified incubator overnight. Next day, the medium was replaced with fresh medium, supplemented with 10 ​mM glycine and cells were further incubated at 37 ​°C and 5 ​% CO_2_ for 24 ​h. In other experiments, cells were seeded in 96-well plates (5,000 ​cell/well), Seahorse XFe24 flux analyzer microplates (5,000 ​cells/well) or glass bottom confocal microscopy dishes (30,000 ​cells/well) and incubated at 37 ​°C in humified incubator overnight.

### Pharmacological modulation of cyanide levels

As characterized in primary hepatocytes and HepG2 hepatoma cells [7], endogenously generated cyanide levels can be suppressed by various cyanide scavengers, including cobinamide and cobalt edetate (0.3–30 ​μM, with 1 ​μM cobinamide yielding maximal inhibitory effects). In the same report, the biochemical pathway of mammalian lysosomal cyanide generation has also been characterized: Lysosomal peroxidases, mainly myeloperoxidase and peroxidasin, catalyze the production of HOCl from H_2_O_2_ and Cl^−^. At physiological lysosomal pH 4.5, glycine is chlorinated by HOCl to generate N,N-dichloroglycine, which spontaneously decomposes into cyanide, CO_2_ and HCl [7]. Thus, mammalian cyanide biogenesis can be suppressed by treating cells with: (a) the broad-spectrum peroxidase inhibitor phloroglucinol (Phl, 1–10 ​μM, 10 ​μM maximally effective); (b) the lysosomal deacidification agent hydroxychloroquine (Hcq, 10 ​μM maximally effective); and (c) serine hydroxymethyltransferase (SHMT) inhibitors – 4,4′-[2,4-pyrimidinediylbis (4,1-phenyleneimino)]bis (4-oxobutanoic acid) (iSHMT) or lometrexol hydrate (LH) – which inhibit serine's conversion to glycine and thus decrease lysosomal glycine (maximally effective at 30 ​μM and 1 ​μM, respectively) [7]. Accordingly, in this study cells were treated with Hcq (10 ​μM), Phl (10 ​μM), iSHMT (30 ​μM), or LH (1 ​μM) for 72 ​h. The cyanide scavenger vitamin B_12_a was used at a concentration of 1 ​μM. Culture medium containing these agents was refreshed every 24 ​h, for a total incubation period of 72 ​h.

### Electrochemical method for cyanide detection

Endogenous cyanide generation in both cell lysates and rat tissue homogenates was quantified using an electrochemical detection method as described [7].

### Confocal microscopy

For confocal microscopy analysis, glass-bottom confocal dishes were coated with poly-ornithine 0.1 ​mg/ml and incubated overnight at 37 ​°C and 5 ​% CO_2_. Dishes were then washed three times with water prior to cell seeding. Detroit 551 and Detroit 539 ​cells were seeded at a density of 30,000 ​cells per well with a total volume of 500 ​μL and incubated overnight at 37 ​°C in a humidified atmosphere containing 5 ​% CO_2_. The following day, cells were treated with cyanide modulators as detailed in the *Cell culture* section. A previously described protocol [7], with a minor modification, has been used to detect intracellular cyanide content by confocal microscopy. Briefly, cells were incubated for 1 ​h with 15 ​μM of the cyanide selective probe (CSP) [12], followed by 10 ​min incubation with 50 ​nM LysoTracker™ Deep Red (cat. L12492 ThermoFisher Scientific/Invitrogen). Cells were then washed 3 times in Hank's Balanced Salt Solution (HBSS). Images were acquired using a Leica SP5 confocal microscope equipped with a 40 ​× ​oil-immersion objective. For each condition, three images per well were captured across three independent biological replicates. Fluorescence intensity was quantified using the ImageJ software [7].

### Cyanide degradation assay

Cyanide degradation assay was used to measure TST activity both from cell lysates and from liver homogenates, as described [7].

### Ion chromatography analysis

Ion exchange chromatography was employed for the determination of sulfite, sulfate, thiosulfate and thiocyanate plasma levels from WT and DS rats. Plasma samples were subjected to protein precipitation using 1:5 ratio of plasma to acetonitrile. The mixture was vortexed for 10 ​s, incubated on ice for 10 ​min and centrifuged at 21,000×*g* for 10 ​min at 4 ​°C. The supernatant was transferred to Si/PTFE conical septum vials.

A 930 Comact IC Flex ion chromatography system (Metrohm, Zofingen, Switzerland) equipped with an 850 Professional IC conductivity detector was used to perform ions quantification. The system included a Metrosep A SUPP5 150/4.0 analytical column and a high throughput autosampler 889 IC sampler Center (Metrohm). For ion analysis, the eluent was composed of 3.2 ​mM Na_2_CO_3_, 1 ​mM NaHCO_3_ and 2.5 ​% acetone. The flow rate was maintained at 0.7 ​ml/min, and the column temperature was set at 35 ​°C. A suppressor regenerant solution made of 100 ​mM ​H_2_SO_4_ was used to reduce background conductivity and enhance sensitivity.

Calibration curves for target ions were constructed using NaSCN, Na_2_S_2_O_3_, Na_2_SO_3_ and Na_2_SO_4_ solutions at concentrations ranging from 0.1 to 6.25 ​mg/l. Quantification was based on peak area and data were processed using the MagIC Net software. Ion concentrations in plasma were reported in μM after correcting for sample dilutions.

### Cell proliferation

Detroit 551 and Detroit 539 ​cells were seeded at a density of 5,000 ​cells per well in a sterile transparent 96-well plate with a total volume of 100 ​μL per well and incubated overnight at 37 ​°C in a humidified atmosphere containing 5 ​% CO_2_. The following day, cells were treated with cyanide modulators as described above. Cell proliferation was monitored as endpoint after 72 ​h using BrdU incorporation assay kit, which was used according to manufacturer's instructions (Merck, 11296736001).

### Western blot analysis

Whole-cell protein extracts (20 ​μg per sample) were resolved on 4–12 ​% Bis-Tris Plus polyacrylamide gels (Invitrogen) and subsequently transferred onto polyvinylidene difluoride (PVDF) membranes using the iBlot 2 dry transfer system (Invitrogen). Membranes were blocked for 1 ​h at room temperature in TBS containing 0.1 ​% Tween-20 (TBS-T) and 5 ​% non-fat dry milk to prevent non-specific binding. Following blocking, membranes were incubated overnight at 4 ​°C with the following primary antibodies, diluted in TBS-T supplemented with 5 ​% bovine serum albumin (BSA): anti-TST rabbit monoclonal antibody (Abcam, ab231248, 1:1,000), anti-LAMP1 rabbit recombinant monoclonal antibody (Abcam, ab225762, 1:1,000), and anti-β-actin mouse monoclonal antibody (AC-15; Sigma-Aldrich, 1:1,000). After overnight incubation, membranes were washed three times with TBS-T and incubated for 1 ​h at room temperature with horseradish peroxidase (HRP)-conjugated secondary antibodies (anti-rabbit IgG or anti-mouse IgG; Cell Signaling Technology, #7076), diluted 1:3,000 in TBS-T containing 5 ​% milk. Subsequently, membranes were washed twice with TBS-T and once with TBS before applying the Radiance Plus Femto HRP substrate (Azure Biosystems, AC2103) for signal development. Chemiluminescent signals were captured using the Azure Imaging System 300 (Azure Biosystems).

### Intracellular glycine determination

Intracellular glycine levels were determined by a fluorometric Glycine Assay Kit, according to manufacturer's instructions (Abcam, ab211100).

### Mitochondrial respiration measurements

Maximal respiratory capacity of dermal fibroblasts (Detroit 539) was measured using the Seahorse XFe24 Flux Analyzer (Agilent Technologies, Santa Clara, CA, USA). Cells were seeded at a density of 5,000 ​cells per well in Agilent Seahorse XF24 ​cell culture microplates, with a total volume of 200 ​μl per well, and incubated overnight at 37 ​°C in a humidified atmosphere containing 5 ​% CO_2_. The following day, cells were treated with cyanide modulators as detailed in the *Cell culture* section. On the day of the assay, the culture medium was replaced with Seahorse XF DMEM supplemented with 2 ​mM glutamine, 1 ​mM pyruvate, and 10 ​mM glucose (final concentrations). Plates were then incubated in a non-CO_2_ incubator at 37 ​°C for 1 ​h to allow for temperature and pH equilibration, following the manufacturer's instructions. The mitochondrial stress test protocol included two baseline measurements of the oxygen consumption rate (OCR), followed by sequential injections of the following compounds: 1 ​μM oligomycin to assess ATP-linked respiration, 2 ​μM FCCP to determine maximal respiratory capacity, and a combination of 0.5 ​μM rotenone and 0.5 ​μM antimycin A to inhibit complexes I and III, respectively, thereby quantifying non-mitochondrial respiration.

The specific activity of mitochondrial complex IV in permeabilized cells was measured using the Extracellular Flux Analyzer, following our previously published protocol [8]. In brief, HepG2 cells were seeded at a density of 20,000 ​cells per well and incubated overnight at 37 ​°C in a humidified atmosphere containing 5 ​% CO_2_. The following day, medium was supplemented with 100 ​μM ​H_2_S releasing donor GYY-4137 (Cayman Chemicals) or vehicle and further incubated for 24 ​h. The next day, cells were incubated for 1 ​h with 30 ​μM KCN and then washed twice with mannitol-sucrose-BSA (MAS-BSA) buffer composed of 70 ​mM sucrose, 220 ​mM mannitol, 10 ​mM KH_2_PO_4_, 5 ​mM MgCl_2_, 2 ​mM HEPES, 1 ​mM EGTA, and 4 ​mg/ml fatty acid-free BSA (pH 7.2). Oxygen consumption rate (OCR) was recorded at steady state and after sequential injections through the instrument's ports. The injection sequence was as follows: Port A – a mixture containing 50 ​μg/ml saponin for cell permeabilization, 0.5 ​mM tetramethyl-*p*-phenylenediamine (TMPD), 2 ​mM ascorbate, 1 ​μM FCCP, and 1 ​mM ADP to stimulate electron flow and respiration; Port B – 1 ​μg/ml oligomycin to inhibit ATP synthase; Port C – 20 ​mM sodium azide to inhibit complex IV and determine non-mitochondrial respiration. All the chemicals for assessing specific activity of mitochondrial complex IV were purchased from Sigma-Merk (Buchs, Switzerland).

In all cases, following completion of the assay, cells were lysed by replacing the assay medium with 20 ​μl of lysis buffer per well. Total protein content was quantified using the BCA assay, and results were normalized accordingly.

### Statistical analysis

Unless otherwise stated, data are presented as mean values ​± ​SEM of several independent experiments where an independent experiment is defined as an experiment performed on a different experimental day, representing a biological replicate (as opposed to technical replicates). No statistical methods were used to pre-determine sample sizes, but our sample sizes are similar to those reported in previous publications [7]. Data distribution was assumed to be normal, but this was not formally tested. Whenever it was logistically possible, data analysis was performed blind to the conditions of the experiments. For instance, the investigator measuring blood cyanide levels was not aware of the designation of the blood samples. No animals data points were excluded. Statistical comparison of two groups was performed using two-sided Student's t-test. Statistical analysis of more than two groups performed by two-way ANOVA followed by post-hoc Bonferroni's test to identify significant differences between two groups. These analyses were performed using Graphpad Prism 8.0 (Boston, MA, USA).

## Results

**Fig. 1:**
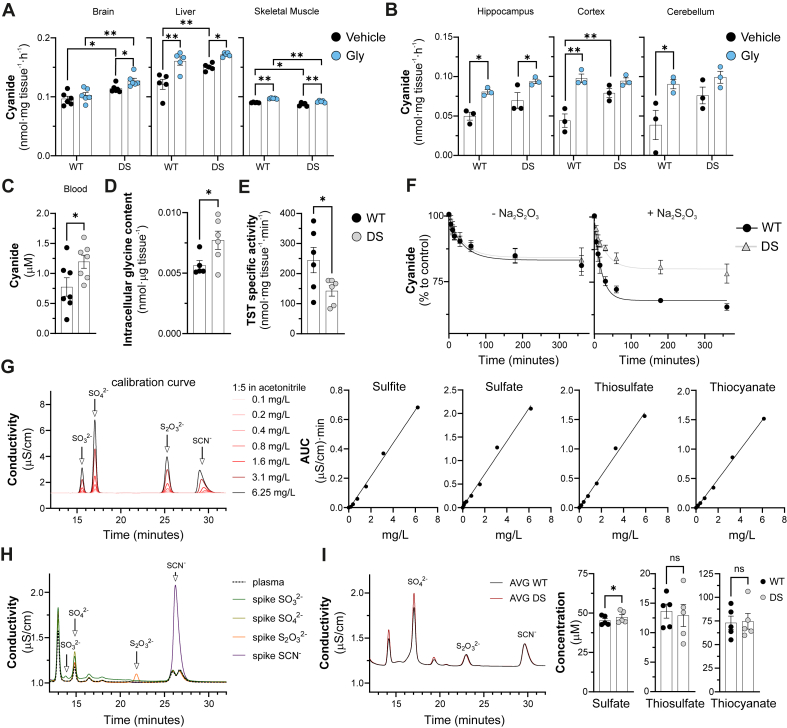
**Cyanide generation is more pronounced in DS rats than in healthy control rats.***(A)* Detection of cyanide generation from brain, liver or muscle homogenates and *(B)* from different brain regions of wild-type control (WT) and DS rats, under basal conditions and in the presence of 10 ​mM glycine, using an electrochemical method. *(C)* Cyanide levels in WT and DS rat blood under baseline conditions. *(D)* Intracellular glycine levels in WT and DS rat brain homogenates. (*E)* TST specific activity of WT and DS rat brain homogenates. (*F*) Thiosulfate and thiocyanate levels in WT and DS rat plasma. (*G*) Calibration curves of sulfite (SO_3_^2−^), sulfate (SO_4_^2−^), thiosulfate (S_2_O_3_^2−^) and thiocyanate (SCN^−^). (*H*) Retention time of ions spiked in human plasma samples. (*I*) Levels of sulfate, thiosulfate and thiocyanate in WT and DS rat plasma. AUC: Area under the curve; ∗p ​≤ ​0.05 and ∗∗p ​≤ ​0.01.

Gently homogenized brains, livers, and skeletal muscles from DS rats [11] generated higher cyanide levels than those from wild-type (WT) controls (Fig. 1A). Among the brain regions, the cortex displayed the most pronounced difference between WT and DS rats (Fig. 1B). DS rats also exhibited elevated blood cyanide levels (Fig. 1C), elevated intracellular glycine content (Fig. 1D), and reduced TST expression [11] and activity (Fig. 1E and F) in brain homogenates. Regarding other related circulating metabolites, DS rats exhibited higher blood sulfate levels, while thiosulfate and thiocyanate levels were comparable in DS rats and wild-type control rats (Fig. 1G–I).

**Fig. 2:**
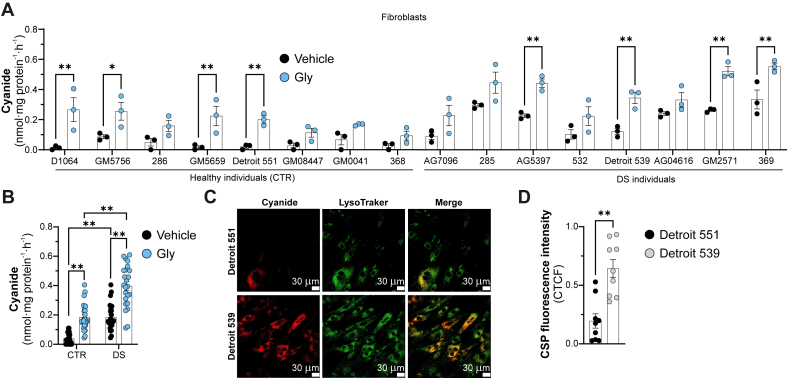
**Cyanide generation in DS fibroblasts is more pronounced than in healthy control fibroblasts.** (*A, B)* Detection of cyanide in a panel of CTR (healthy) and DS human fibroblasts in normal medium and after supplementation with 10 ​mM glycine for 24 ​h using an electrochemical method. *(C, D)* Cyanide detection in healthy (Detroit 551) and DS (Detroit 539) human fibroblasts using confocal microscopy. ∗p ​≤ ​0.05 and ∗∗p ​≤ ​0.01.

Human DS fibroblasts also exhibited higher baseline cyanide production than control fibroblasts. Moreover, the addition of glycine induced a more pronounced stimulation of cyanide generation in DS cells than in control cells (Fig. 2A). Despite a considerable variability among individual cell lines, in aggregate, DS cells consistently exhibited higher basal and glycine-stimulated cyanide production than control cells (Fig. 2B). We further characterized cyanide overproduction by comparing a healthy control fibroblast line (Detroit 551) with a DS fibroblast line (Detroit 539). Confocal imaging confirmed higher cyanide levels in DS cells, with the signal localized most intensively to the lysosomes, but also detected in the cytosol (Fig. 2C and D).

**Fig. 3:**
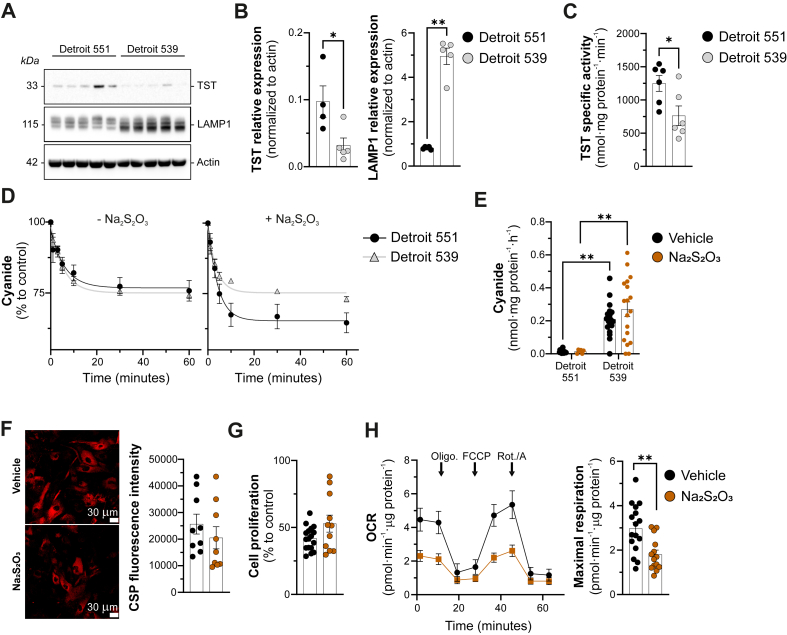
**Increased cyanide generation in DS fibroblasts is associated with an impairment of TST-mediated cyanide catabolism.***(A, B)* Representative western blots and quantification analysis of TST and Lamp1, respectively. *(C, D)* TST-specific activity of Detroit 551 and Detroit 539 fibroblasts. *(E)* Cyanide detection from healthy (Detroit 551) and DS (Detroit 539) human fibroblasts ​± ​100 ​μM sodium thiosulfate (Na_2_S_2_O_3_), for 72 ​h using the electrochemical cyanide detection method. *(F)* Cyanide production from Detroit 539 patients derived fibroblasts ​± ​100 ​μM sodium thiosulfate (Na_2_S_2_O_3_), for 72 ​h using confocal microscopy. *(G)* Cell proliferation rate (BrdU incorporation) and *(H)* mitochondrial maximal respiration in Detroit 539 fibroblasts ​± ​100 ​μM sodium thiosulfate (Na_2_S_2_O_3_), for 72 ​h ∗p ​≤ ​0.05 and ∗∗p ​≤ ​0.01.

Lysosomal Associated Membrane Protein 1 (LAMP1) expression was higher in DS fibroblasts than in controls indicative of increased lysosomal size and number in DS (Fig. 3A–C), consistent with previous reports [13,14]. TST expression and activity were markedly reduced in DS cells compared to controls (Fig. 3C and D): when stimulating TST with its physiological co-factor thiosulfate, cyanide's conversion to thiocyanate was accelerated in the control cells but was only minimally affected in DS cells, indicating low TST activity in DS cells (Fig. 3D). Additionally, treatment with thiosulfate did not reduce cyanide levels in DS cells (Fig. 3E and F), did not alter the proliferation rate of DS cells (Fig. 3G) and in fact it impaired cellular bioenergetic function (Fig. 3H).

**Fig. 4:**
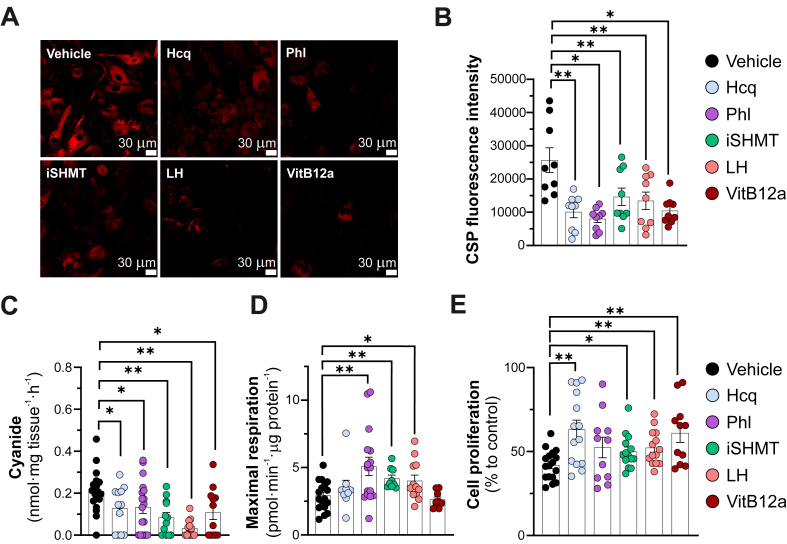
**Cyanide toxicity in Detroit 539 DS-fibroblasts is reduced by long-term treatment with various cyanide modulators.***(A–C)* Detection of cyanide generation from DS (Detroit 539) cells treated for 72 ​h with vehicle or with hydroxychloroquine (Hcq, 10 ​μM), phloroglucinol (Phl, 10 ​μM), the serine hydroxymethyltransferase inhibitor iSHMT (30 ​μM), the serine hydroxymethyltransferase inhibitor lometrexol hydrate (LH, 1 ​μM) or vitamin B12 (VitB12a, 1 ​μM) using confocal microscopy *(A,B)* or the electrochemical detection method *(C)*. *(D)* Mitochondrial maximal respiration and *(E)* cell proliferation rate (BrdU incorporation) in Detroit 539 fibroblasts ​± ​10 ​μM Hcq, 10 ​μM Phl, 30 ​μM iSHMT, 1 ​μM LH or 1 ​μM VitB12a, for 72 ​h ∗p ​≤ ​0.05 and ∗∗p ​≤ ​0.01.

Pharmacologically suppressing lysosomal cyanide generation partially improved the bioenergetics and/or proliferation of DS cells (Fig. 4D and E). This was achieved using several approaches: lysosomal deacidification with hydroxychloroquine (shifting lysosomal pH away from the optimal range for cyanide production), peroxidase inhibition with phloroglucinol (reducing HOCl formation required for cyanide generation), inhibition of serine hydroxymethyltransferase (via two different SHMT inhibitors to lower intracellular and lysosomal glycine concentrations) [7], or direct cyanide scavenging with vitamin B_12_a (hydroxocobalamin) (Fig. 4A–C). The partial nature of these metabolic and proliferative improvements may be explained, at least in part, by the irreversible nature of CCOx inhibition by cyanide [4]. Mitochondrial electron transport can only be fully restored after *de novo* synthesis of Complex IV, which likely takes longer than the 72 ​h duration of our experiments.

**Fig. 5:**
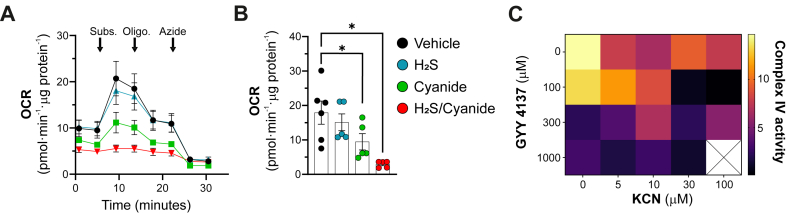
**Cumulative toxicity of cyanide and H_2_S on Complex IV (CCox) activity in HepG2 cells.***(A, B)* Inhibition of mitochondrial on Complex IV activity by cyanide (generated from 30 ​μM KCN), H_2_S (generated from 100 ​μM GYY-4137). ∗p ​≤ ​0.05 and ∗∗p ​≤ ​0.01. *(C)* Heat map showing the effect of various concentrations of the two molecules on CCox activity.

Another reason may be related to the presence of high levels of H_2_S in the DS cells. Previous studies demonstrated that the mitochondrial suppression in DS is related to the overproduction of hydrogen sulfide (H_2_S), resulting from gene dosage effects due to cystathionine beta-synthase (CBS) being encoded on Chromosome 21 [2,10,11]. Indeed, CBS inhibition or siRNA-mediated downregulation of CBS improves cellular bioenergetics, proliferation, and neurocognition in DS models [2,10,11]. Given that cyanide and H_2_S both inhibit mitochondrial respiration via CCOx [4], we investigated their combined effect on cellular bioenergetics. Low concentrations of cyanide (generated from its potassium salt) partially inhibited CCOx activity, but simultaneous administration of low, non-inhibitory concentrations of H_2_S (generated via administration of 100 ​μM of the slow-release donor GYY4137) [15]) together with an intermediate concentration of cyanide (30 ​μM) caused a near-complete inhibition of CCOx activity, indicative of a cumulative action (Fig. 5A and B). Additive effects of the two donors were also noted at many other GYY4137/cyanide combinations (e.g. 100 μM/100 ​μM; 1000 μM/5 ​μM, 1000 μM/10 ​μM and 1000 μM/30 ​μM). In contrast, at 300 ​μM GYY4137, where its CCOx inhibitory effect was very pronounced, addition of cyanide did not produce any further inhibition (Fig. 5C). These findings suggest that co-presence of cyanide and H_2_S can cooperatively suppress metabolism via combined CCOx inhibition.

## Discussion

Low levels of cyanide were found to be produced in the brain and in neuronal cell lines in several reports published in the early 2000's [5,6]. It was also suggested that endogenous cyanide generation in neurons may involve a peroxidase mechanism [5,6,[16], [17], [18], [19]]. Recent work has provided new insights into cyanide biogenesis and its physiological role. It demonstrated that **(a)** the lysosome is the source of endogenous cyanide in mammalian cells; **(b)** low levels of cyanide support normal cell function and confer bioenergetic regulation and cytoprotection; and **(c)** higher cyanide levels (for example, upon TST silencing or impaired glycine degradation leading to glycine accumulation) cause mitochondrial suppression [7].

By demonstrating increased cyanide levels in DS, the current study places the above observations into a pathophysiological contex. Our data demonstrate that **(a)** DS rats have elevated blood cyanide levels; **(b)** DS tissues generate more cyanide than wild-type controls under both basal conditions and after glycine supplementation; **(c)** human DS fibroblasts produce higher cyanide levels than healthy control cells; and **(d)** this cyanide overproduction in DS is associated with a marked downregulation of TST (rhodanese), the key enzyme responsible for cyanide detoxification. Furthermore, we found that **(e)** suppressing lysosomal cyanide production in DS cells (through cyanide scavengers, lysosomal deacidification, or inhibition of serine/glycine conversion) improves cellular bioenergetics and enhances proliferation rates. Finally, **(f)** we provide evidence for a cooperative interaction of cyanide and H_2_S at the level of CCOx inhibition, and hypothesize that this combined action may contribute to the DS-associated metabolic suppression.

In this study, we used various pharmacological agents to modulate cellular cyanide levels: a cyanide scavenger (vitamin B_12_a), a peroxidase inhibitor (phloroglucinol), a lysosomal deacidification agent (hydroxychloroquine), and two different SHMT inhibitors. If increased cyanide production is directly responsible for reduced mitochondrial ATP generation and cell proliferation in DS cells, then all of these cyanide-lowering interventions would be expected to produce similar, proportional improvements in cellular metabolism and proliferation. Indeed, as shown in Fig. 4, this was the case for both SHMT inhibitors, and similar effects were observed with the peroxidase inhibitor phloroglucinol, which improved cellular respiration and also tended to increase cell proliferation. However, vitamin B_12_a and hydroxychloroquine, despite lowering cyanide levels, did not significantly improve maximal respiration; nonetheless, they did enhance cell proliferation rates. One explanation for these findings is that **(a)** the relationship between aerobic ATP production and cell proliferation is complex and influenced by many factors, and **(b)** hydroxychloroquine and vitamin B12a have multiple pharmacological effects beyond cyanide modulation.

The exact mechanism by which endogenous cyanide levels are elevated in DS cells and tissues remains to be further characterized. In the current report we demonstrate that TST protein levels are markedly lower (approx. 25 ​% of control levels) in the DS cells utilized in the current project, compared to control fibroblasts. Similarly, in a prior study, utilizing a broad selection of human DS fibroblasts, we have observed, on average, an approximately 40 ​% decrease in TST expression in DS cells, with a high individual variability [2]. This degree of individual variability is not uncommon in DS, as different individuals often compensate for the extra chromosome 21 by differentially regulating genes on other chromosomes [3,[20], [21], [22]]. Likewise, DS rats show lower TST expression than wild-type controls, with considerable variability among individuals and across brain regions [11]. Thus, based on data showing that TST knockdown markedly elevates cyanide levels and causes metabolic suppression in hepatocytes [7], the downregulation of TST in DS is likely a major contributor to the elevated cyanide levels. However, changes in TST expression and activity in DS likely vary between individuals and may differ by organ and even by region within organs (e.g., across different brain regions). We hypothesize that this variability leads to corresponding differences in cyanide clearance capacity, resulting in significant individual- and region-specific differences in cyanide levels in DS.

The exact mechanism by which TST protein is downregulated and TST activity is increased in DS remains to be further investigated. The TST promoter contains redox-sensitive elements [23], and it is conceivable that the redox imbalance in DS may affect the activation of the TST promoter. However, recent meta-analysis, demonstrating that in DS cells and tissues TST mRNA levels are not significantly altered [14] speaks against a transcriptional mechanism. Thus, alterations at the level of TST stability/degradation may underlie this phenomenon and this possibility should be explored in future studies. Based on the data presented in the current report, pharmacological activation of TST with thiosulfate does not appear to be a viable experimental therapy in DS, as thiosulfate in DS cell homogenates does not accelerate cyanide degradation (Fig. 3E), and suppresses mitochondrial respiration (Fig. 3H). The latter effect may be due to the well-documented intracellular conversion of thiosulfate to H_2_S [24], since high concentrations of H_2_S –– especially in DS cells [2,10,11,25] –– are known to suppress cell respiration.

However, additional mechanisms –– e.g. an increased cyanide generation in DS lysosomes –– may also play a role. Indeed, we have observed a markedly higher levels of the lysosomal marker LAMP-1 in DS fibroblasts compared to control (suggestive of lysosomal hypertrophy), and several prior reports demonstrate that DS lysosomes exhibit significant functional alterations in DS [13,14,26,27]. Indeed, our confocal imaging data show a markedly higher lysosomal CN signal in DS cells compared to control cells (Fig. 3A and B). Since lysosomes are the source of cyanide, and posttranslational cyanide modifications (protein cyanylation) are most pronounced in the lysosomal compartment [7], it is conceivable tha cyanide modulates, perhaps impairs various metabolic and signalling functions of the DS cell. This possibility remains to be investigated in the future.

Other factors may also be altered in DS, such as intracellular/lysosomal glycine levels, peroxidase expression or activity, and oxidant (hypochlorous acid) levels. Since all of these are components of the glycine-to-cyanide pathway, their dysregulation could contribute to elevated cyanide production in DS. Indeed, we have detected higher levels of glycine in DS cells than in control cells (Fig. 1D) and in some –– but not all –– prior reports, DS was found to be associated with elevated glycine levels [[28], [29], [30], [31]]. Likewise, there are prior reports demonstrating or suggesting increased peroxidase activity and/or increased hypochlorous acid levels in DS cells and tissues [5,32].

Confocal imaging shows that in DS cells cyanide not only localizes to the lysosomes, but it also is abundantly detectable in all intracellular components (Fig. 2C). In mitochondria, cyanide is known as a potent inhibitor of Complex IV (cytochrome *c* oxidase), and this effect is primarily responsible for its inhibitory effect on cellular metabolism and viability [6]. However, additional actions may also contribute to cyanide's adverse cellular effects; cyanide is known to induce intracellular calcium overload via calcium-channel opening and mobilization of calcium from intracellular pools. Cyanide can activate NMDA receptors [19], which may also contribute to its neurotoxic effects via inducing or potentiating excitotoxic neuronal damage. In addition, Complex IV inhibition – in part via the ATP depletion it causes – can trigger further downstream damage. These effects include increased reactive oxygen species (ROS) production (due to inhibition of antioxidant enzymes like glutathione peroxidase and superoxide dismutase), caspase activation, endoplasmic reticulum stress, microtubule disorganization, and even dysregulation of gene transcription [6,[33], [34], [35], [36], [37], [38], [39]]. These actions may, in turn, create various deleterious cellular feedforward cycles of neuroinjury and/or impaired neurodevelopment in DS. Indeed, low-level chronic cyanide exposure is known to cause neurocognitive and neurodevelopmental deficits *in vivo* [[39], [40], [41]]. Since our data suggest that cyanide overproduction is a systemic response in DS, cyanide may also contribute to deficits and dysfunctions in various organ systems in DS outside the CNS.

Importantly, the current report also shows an increase in circulating (whole blood) cyanide levels in DS as well (Fig. 1C). In the circulation, cyanide is predominantly present in the red blood cells (i.e. not in the plasma), and it is bound to hemoglobin [[42], [43], [44], [45]]. The primary mode of the underlying reaction involves the reaction of cyanide with methemoglobin to form cyanomethemoglobin [46,47]. Thus, circulating cyanide is predominantly present in a bound, biologically inactive form (released upon acidification and subsequently detected by our cyanide assay [7]. The above considerations explain how the body can ‘tolerate’ circulating cyanide. This also addresses a potential criticism of the current study, which is that cigarette smoke contains high levels of cyanide and the blood of human smokers contains high blood levels of cyanide [[48], [49], [50], [51]]. The key difference is that in smokers, inhaled cyanide enters the bloodstream and immediately binds to hemoglobin, sequestering it in an inactive pool. By contrast, in DS (and other conditions of endogenous cyanide overproduction), cyanide is produced intracellularly, giving it immediate direct access to intracellular targets.

Interestingly, in our *in vivo* DS model, the higher circulating cyanide levels were also associated with higher sulfate levels, whereas thiosulfate and thiocyanate levels were comparable in DS and wild-type animals (Fig. 1I). In the canonical H_2_S catabolic pathway sulfite has two possible fates: **(a)** via the TST route => conversion to thiosulfate and **(b)** via the sulfite oxidase (SUOX) route => conversion to the more stable metabolite, sulfate [53]. In DS, where TST expression and activity is reduced, it is conceivable that the SUOX route is favored, which would be associated with an increase of sulfate levels (which is indeed the case). Likewise, the reduced TST activity may explain why the increased blood cyanide levels in DS are not also associated with increased thiocyanate levels. With respect to circulating thiosulfate levels in DS, at least two factors may be involved: **(a)** the action of TST, which primarily synthesizes, rather than consumes thiosulfate [53]: in DS, since TST activity is reduced, a *reduction* in thiosulfate levels would be expected and **(b)** the increased expression of CBS and the increased production of H_2_S [14], which would be expected to *increase* thiosulfate levels. The lack of change in DS, as observed in our i*n vivo* experiments, may be the combined consequence of these two influences.

In addition to TST, a second mammalian enzyme, 3-mercaptopyruvate sulfurtransferase (3-MST) also has the ability to metabolize cyanide by converting it (along with 3-mercaptopyruvate) into pyruvate and thiocyanate [54]. Interestingly, this enzyme –– although not encoded on chromosome 21 –– is also known to become upregulated in human DS cells [2,55] and in DS animal models [11,56]. This upregulation shows significant variability among individuals [2,55] and across brain regions [11]. The presence and upregulation of this enzyme in DS likely add additional complexity and variability to cyanide level regulation. The role of 3-MST in modulating endogenous cyanide levels (both generally and in DS specifically) remains to be investigated.

Prior studies have demonstrated that DS is associated with a suppression of mitochondrial Complex IV (CCox) activity, and in DS cells and tissues the aerobic, mitochondrial, physiological pathway of ATP generation is suppressed; in turn, cells upregulate their glycolytic activity in a process termed pseudohypoxia [2,3]. The gaseous mammalian mediator H_2_S has been implicated in the suppression of mitochondrial Complex IV activity in DS cells [14,52], and inhibition of H_2_S generation was found to improve cellular metabolism in DS cells *in vitro* [2,14,25,[55], [56], [57], [58]] and neurocognition in animal models of DS *in vivo* [11,56]. Moreover, clinical observations demonstrate that DS is associated with an elevation of urinary thiosulfate levels in several (but not all) available reports (as reviewed in Ref. [10]). The current report demonstrates that endogenously generated cyanide may also contribute, and in fact there may be a cooperative interaction between these two biological gases. Both cyanide and H_2_S have the same target, CCox, and both of them are thought to bind to the same target, the ferric (Fe^3+^) heme a_3_-Cu_B binuclear center, with the effect of H_2_S being reversible, while the effect of cyanide being predominantly irreversible [4]. Thus, when cyanide and H_2_S are both present in a system, the effect of cyanide –– which has approximately 10–30 times higher affinity to Complex IV than H_2_S [4,59,60], and is an irreversible inhibitor, is expected to predominate. However, our experimental data suggest a more complex, concentration-dependent interaction, with additive effects especially at lower concentrations of each mediator. Recent work demonstrates that the inhibition of Complex IV by H_2_S is more complex than previously believed, because, as, at low concentrations –– which are lower than the concentrations necessary to inhibit cytochrome *c* oxidase –– H_2_S also can act as a reductant for cytochrome *c* oxidase [59]. There are currently no experimental data or modelling studies regarding the combined effect of cyanide and H_2_S on isolated cytochrome *c* oxidase. Nevertheless, the cell-based functional test we have conducted –– where, admittedly, each of the two gases may also exert effect on various other cellular targets, which, then, may exert secondary effects on mitochondria –– suggests a complex mode of interaction, including a cumulative/additive inhibitory effect, perhaps via a H_2_S-mediated ‘priming’ of a cyanide-mediated inhibition.

Clearly, further studies are needed to fully understand the consequences of elevated cyanide in DS pathophysiology. Nevertheless, our findings suggest that pharmacologically mitigating cyanide production or action may be a useful experimental therapeutic strategy in DS. Since the effect of cyanide on mitochondrial Complex IV is irreversible, relatively long treatment durations may be necessary, allowing time for *de novo* mitochondrial complex resynthesis under chronically reduced cyanide conditions. The approaches exemplified in the current report include lysosomal deacidification agents (e.g. hydroxychloroquine, which is a clinically used compound, in some instances, also used in long-term administration) [61,62], and/or various glycine transporter inhibitors, which are in clinical trials [63,64], and/or cyanide scavengers, some of which are vitamins that can be administered at high doses (e.g. Vitamin B12a) [65,66] and some of which are clinically used drugs (e.g. dicobalt edetate, an antidote for cyanide poisoning) [[67], [68], [69]]. Sodium thiosulfate is also used as a cyanide antidote –– primarily by stimulating TST [[67], [68], [69]] –– but the data presented in the current study do not support its potential utility in DS. The possibility of combined inhibition of H_2_S generation (e.g. with pharmacological inhibitors of CBS) with cyanide neutralization strategies may also be considered in the future.

## Data availability

All data are available upon reasonable request from the corresponding author.

## Author contributions

Csaba Szabo initiated and conceptualized the study. Maria Petrosino, Karim Zuhra, Anna Kieronska-Rudek, Lucia Janickova, Olivier Bremer, and Moustafa Khalaf conducted experiments. Brian A. Logue, Csaba Szabo and Karim Zuhra provided resources. Maria Petrosino, Karim Zuhra Brian A. Logue and Csaba Szabo drafted and wrote the paper.

## Funding

This work was supported by a “Pool” grant from the University of Fribourg to C.S, the Swiss National Science Foundation Spark CRSK-3_221109 grant to K.Z. and funding from South Dakota State University to B.L.

## Declaration of Competing Interest

M.P., K.Z. and C.S. are inventors on a patent filing related to various cyanide neutralization approaches in Down syndrome.
