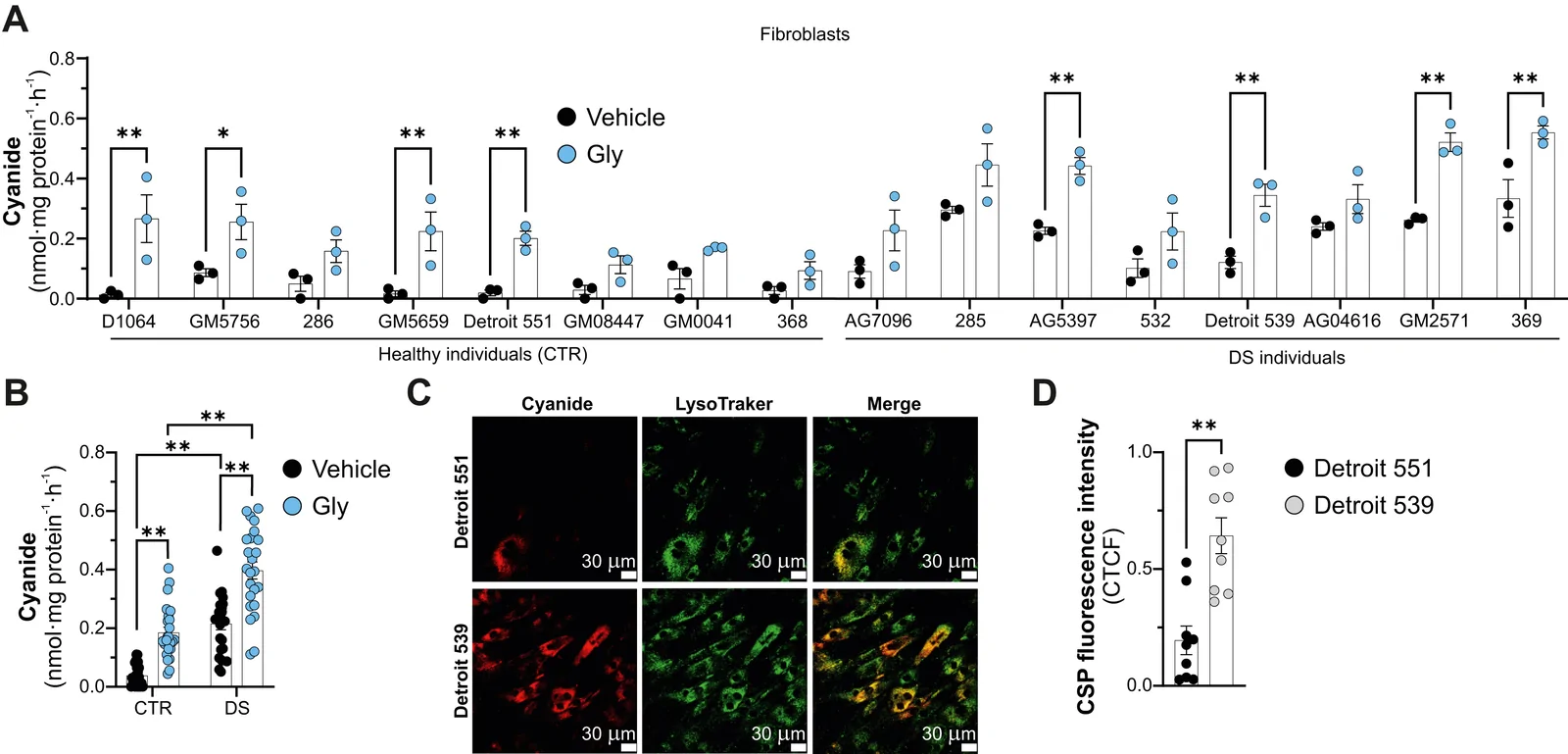# Corrigendum to “Cyanide overproduction impairs cellular bioenergetics in Down syndrome”

**DOI:** 10.1016/j.neurot.2026.e01116

**Published:** 2026-10-30

**Authors:** Maria Petrosino, Karim Zuhra, Anna Kieronska-Rudek, Lucia Janickova, Olivier Bremer, Moustafa Khalaf, Brian A. Logue, Csaba Szabo

**Affiliations:** a Section of Pharmacology, Department of Oncology, Microbiology and Immunology, Faculty of Science and Medicine, University of Fribourg, Fribourg, Switzerland; b Department of Chemistry, Biochemistry and Physics, South Dakota State University, Brookings, SD, USA

The authors regret that the original version of this article contained an error in Figure 2B. During preparation of the pooled dataset, the 24 values for the healthy control + glycine group were inadvertently copied into the Down syndrome (DS) vehicle group, resulting in the duplication of those data points in the published panel.

The correct DS vehicle values were already presented in Figure 2A, which displays the corresponding individual fibroblast cell lines. Figure 2B has now been reconstructed using these original values; the remaining three groups are unchanged. The mean basal cyanide production in DS fibroblasts increases from 0.1849 to 0.2150 nmol/mg protein/h. Reanalysis by two-way ANOVA followed by Bonferroni post-hoc comparisons confirms that all four comparisons indicated in Figure 2B remain significant (p < 0.01). Figure 4C was checked and contains the correct DS vehicle data; it is unaffected by this error.

The corrected Figure 2 is provided below. The authors confirm that this correction does not change the principal findings or conclusions of the work.

**Figure:**